# Risk of primary Sjogren’s Syndrome following human papillomavirus infections: a nationwide population-based cohort study

**DOI:** 10.3389/fimmu.2022.967040

**Published:** 2022-08-16

**Authors:** Huang-Hsi Chen, Kevin Sheng-Kai Ma, Chen Dong, Wen-Jung Chang, Kuan-Rong Gao, Wuu-Tsun Perng, Jing-Yang Huang, James Cheng-Chung Wei

**Affiliations:** ^1^ Division of Allergy, Immunology, and Rheumatology, Chung Shan Medical University Hospital, Taichung, Taiwan; ^2^ Institute of Medicine, Chung Shan Medical University, Taichung, Taiwan; ^3^ Center for Global Health, Perelman School of Medicine, University of Pennsylvania, Philadelphia, PA, United States; ^4^ Department of Epidemiology, Harvard T.H. Chan School of Public Health, Boston, MA, United States; ^5^ Graduate Institute of Biomedical Electronics and Bioinformatics, National Taiwan University, Taipei, Taiwan; ^6^ Department of Obstetrics and Gynecology, Dajia Lees General Hospital, Taichung, Taiwan; ^7^ Department of Obstetrics and Gynecology, Yuanli Lees General Hospital, Miaoli, Taiwan; ^8^ National Pingtung University of Science and Technology, Department of Recreational Sport & Health Promotion, Pingtung, Taiwan; ^9^ Institute of Medicine, College of Medicine, Chung Shan Medical University, Taichung, Taiwan; ^10^ Graduate Institute of Integrated Medicine, China Medical University, Taichung, Taiwan

**Keywords:** Human papillomavirus, infection, autoimmunity, primary Sjogren's Syndrome, cohort study

## Abstract

**Introduction:**

Viral infection is an exogeneous factor for primary Sjogren’s syndrome (pSS). This study investigated the association between human papillomavirus (HPV) infections and pSS through a nationwide population based cohort study.

**Methods:**

Patients with HPV infections between January, 1999 and December, 2013 were included. The incidence of new-onset pSS in patients with HPV infections and non-HPV controls were derived. The multiple Cox regression model derived the risk of pSS in patients with HPV infections. Subgroup analysis and sensitivity analysis were performed to validate the association.

**Results:**

During a follow-up period of 12 years, the adjusted hazard ratio (aHR) of pSS in patients with HPV infections was significantly higher than that in non-HPV controls (aHR=1.64, 95% CI=1.47-1.83, P<0.001). The risk of pSS increased with age and the risk increased by 2.64-fold (95% CI= 2.37-2.93) for those older than 45 years. The significant association between HPV infections and the risk of pSS persisted in the sensitivity analysis restricted in HPV infections that lasted over 12 months (aHR=1.63, 95%CI=1.45-1.83, P<0.0001). Subgroup analyses revealed that both male (aHR=1.83, 95%CI=1.47-2.28, P<0.0001) and female (aHR=1.58, 95%CI=1.40-1.79, P<0.0001) patients with HPV infections and HPV-infected patients aged between 16 and 45 years (aHR=1.60, 95%CI=1.34-1.91, P<0.0001) and over 45 years (aHR=1.67, 95%CI=1.46-1.91, P<0.0001) were associated with a significantly greater risk of pSS.

**Conclusion:**

Patients with HPV infections presented with a significantly higher risk of pSS, regardless of age and sex.

## Introduction

Primary Sjogren’s syndrome (pSS) is a chronic autoimmune disorder manifested as lymphocytic infiltration, which causes inflammation of the exocrine glands. The destructed glands result in dryness of the eyes and mouth, pain of the joints and fatigue (1). 30 to 40% of pSS patients have other systematic complications involving lungs, kidneys, gastrointestinal (GI) tract, and nervous system. Epidemiological data has revealed that pSS prevalence is approximately 1% of general population and female predominance (16:1) (2). Genetic inheritance and environmental factors are previously reported to enhance pSS pathogenesis; however, pSS etiology remains unclear, for which viral infection has been as well considered an external factor for it induces chronic inflammation (3, 4). Viral particles translated by viral gene would act as autoantigens, which attract auto-antibodies to combine and trigger proliferation of B cells to cause autoimmune disease. Infected cells may also activate cytotoxic T cells in response through the major histocompatibility (MHC) class I (MHC-1) antigen presentation pathway. Various virus including human T-lymphocytic virus type-1 and type-5, Epstein-Barr virus, Coxsackie virus play an important role in activating auto-inflammation. Several other infection agents including human herpes virus 6, human immunodeficiency virus, and hepatitis B have also been reported to trigger pSS (3, 5). The potential associated mechanisms between viral infections and the immune-pathogenesis of pSS require further research.

Human papillomaviruses (HPVs) are transmitted through sexual intercourse or direct contact with macerated skin and they exclusively invade the epithelium and mucous membranes (6). The HPV prevalence in Taiwan is about 10-20% (7) and predominately in women, which corresponds to the prevalence of pSS in female sex. HPV 16 and 18 are the most prevalent types in female population in Taiwan and other Asian countries (8, 9). There are two prophylactic HPV vaccines available in Taiwan currently. The bivalent vaccine protects against HPV type 16 and 18 and young women before sexual debut are recommended to be vaccinated. The quadrivalent vaccine targets HPV type 6, 11, 16, and 18 (10), and both genders are recommended to be vaccinated (11, 12). Taiwan government supports HPV vaccination, but less than 4% of female population has been vaccinated (12). More than 80% of Taiwanese females are still susceptible to HPV infection due to lack of health education.

A recent study indicates that female patients with rheumatic diseases have higher risk for HPV infections, which might be due to disarranged immune system or immunosuppressive effect of treatments (8). However, currently there is no evidence that supports a reverse correlation of the incidence of pSS in HPV-infected patients, despite an autoimmune phenotype observed in HPV patients (13). As such, the present study aims to determine the incidence of pSS in patients with HPV infections.

## Methods

### Data source

We used the Longitudinal Health Insurance Database 2000 (LHID2000) as our data source, which is a subset of the National Health Insurance Research Database (NHIRD) (14, 15). The LHID2000 contains information on one million randomly selected patients’ sex, date of birth, clinical interventions, hospitalization, medications, dosages, and diagnoses in accordance to the requirements of the International Classification of Disease–Ninth Revision, Clinical Modification, from 2000 to 2013. Demographic dates, records of inpatient or outpatient visits, catastrophic illness, items of medical expenditure, and other clinical materials were available in this database and extracted for research purpose of this study. With a large sample size, the database has been largely used in observational studies (16). The research protocol of the present study was approved and supervised by the Institutional Review Board (IRB) of Chung Shan Medical University Affiliated Hospital.

### Study design

To clarify the association between HPVs and the risk of pSS, we designed a retrospective cohort study. The exposure group and the controlled group were followed up from the index date to the date of pSS onset, study subjects withdrawal, or study completion. The index date was defined as the date of HPV diagnosis. The observational period was set between January, 1999 and December, 2013. We also selected several comorbidities to investigate their effects on the incidence of pSS. The HPV group included patients with new-onset HPV infections between 2002 and 2012; while the non-HPV group included patients who did not have HPV infections during the study period. Propensity score matching of 1:4 by sex, year of birth and the index date was used to select non-HPV controls. For both cases and controls, individuals of any missing demographic data, patients who developed pSS before index date, aged below 16 in 2002, or had HPV infections before 2002 and new-onset HPV infections after 2012, were excluded.

### Study population

**Figure 1:**
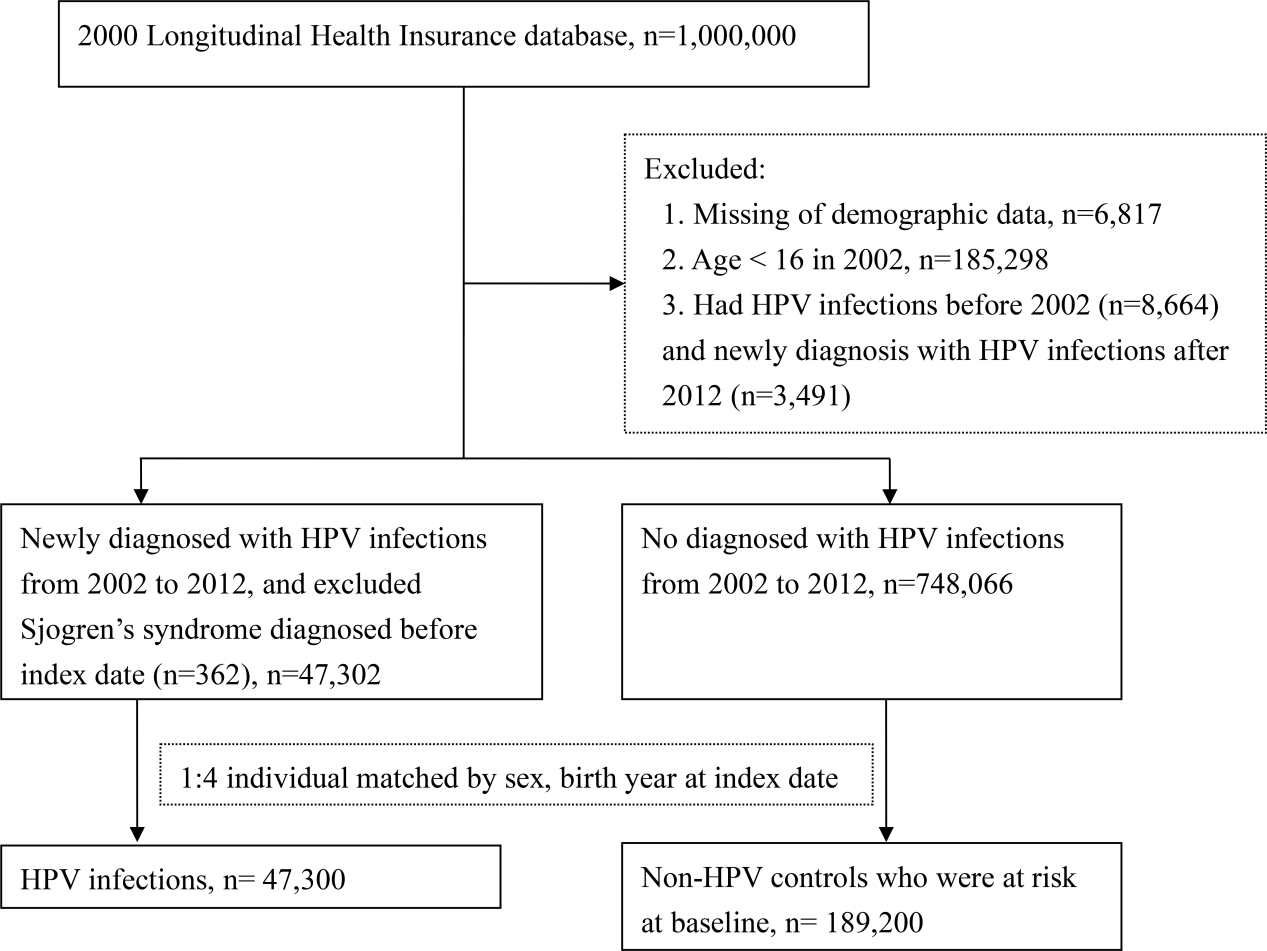
Flowchart for study design.

After excluded 362 patients with pSS diagnoses prior to HPV infections, 6,817 patients with missing demographic data, 185,298 patients younger than 16 years in 2002, 8,664 patients with previous HPV diagnoses before 2002, and 3,491 patients with HPV diagnoses after 2012, a total of 47,302 patients with first HPV diagnoses (ICD-9 code: 078.11, 079.4, 078.1, 078.10–078.12, 078.19, 759.05, 795.09, 795.15, 795.19, 796.75 and 796.79, along with records of positive polymerase chain reaction test results) between 2002 and 2012 were enrolled in the HPV group (**Figure 1**).

748,066 patients without HPV diagnoses between 2002 and 2012 were enrolled as the comparison group and matched to patients in the HPV group by sex, year of birth and the index date at a ratio of 1:4. As a result, there were 47,300 patients in the HPV exposure group and 189,200 matched-controls in the control group at baseline (**Figure 1**).

### Variables and measurements

New-onset pSS was set as the primary endpoint of this study and the diagnoses were based on the American College of Rheumatology (ACR)-the European League Against Rheumatism (EULAR) classification criteria (17). To ensure the consistency of the diagnoses, the diagnostic records of pSS have to be confirmed by at least 3 outpatient records within 2 years or at least 1 hospital admission history between 2002 and 2012. Predictor variables included comorbidities such as rheumatoid arthritis, pneumonia, bronchitis, dental caries, chronic liver disease, cholelithiasis, interstitial nephritis, calculus of kidney, urinary tract infection, arthralgia, chronic obstructive lung disease (COPD), as identified within 2 years preceding the index date.

### Statistical analysis

Propensity score matching (18–25) was used to homogenize the baseline characteristics of the two groups (26–35). To reveal the potential risk factors contributing to the pathogenesis of pSS during follow-up, we performed multiple Cox regression on selected comorbidities to estimate the adjusted hazard ratios (aHRs) of pSS and calculated the corresponding 95% confidence intervals (CIs) to ensure the precision and accuracy of the analysis. We recognized statistically significant results with a two-tailed p value <0.05. All data were analyzed using SAS software (version 9.4; SAS Institute, Inc., Cary, NC, USA).

## Results

### Baseline characteristics

In total, 47,300 HPV-infected patients and 189,200 non-HPV individuals were enrolled at the baseline. The mean age at the time of diagnosis of HPV was 42.59 ± 16.43 years, and 59.24% of HPV patients were between the age of 16 and 45. The female to male ratio was slightly greater than 1 (50.03% vs. 46.97%). After propensity score matching, baseline demographics and comorbidities were balanced (**Table 1**).

**Table 1:** Characteristics in study groups at baseline.

	HPV infections	Non-HPV controls	p value
	n = 47,300	n = 189,200	
Age at baseline, Mean ± SD	42.59 ± 16.43	42.59 ± 16.43	1.0000
16-45	28,021 (59.24%)	112,084 (59.24%)	
≧45	19,279 (40.76%)	77,116 (40.76%)	
Sex			1.0000
Female	25,083 (53.03%)	100,332 (53.03%)	
Male	22,217 (46.97%)	88,868 (46.97%)	
Comorbidities			
Rheumatoid arthritis	404 (0.85%)	1,179 (0.62%)	<.0001
Pneumonia	1,468 (3.10%)	5,022 (2.65%)	<.0001
Bronchitis	1,914 (4.05%)	5,478 (2.90%)	<.0001
Dental caries	23,425 (49.52%)	69,654 (36.82%)	<.0001
Chronic liver disease	5,100 (10.78%)	14,246 (7.53%)	<.0001
Cholelithiasis	905 (1.91%)	2,667 (1.41%)	<.0001
Interstitial nephritis	958 (2.03%)	2,786 (1.47%)	<.0001
Calculus of kidney	1,419 (3.00%)	4,143 (2.19%)	<.0001
Urinary tract infection	6,924 (14.64%)	21,431 (11.33%)	<.0001
Arthralgia	5,545 (11.72%)	18,085 (9.56%)	<.0001
COPD	4,418 (9.34%)	13,588 (7.18%)	<.0001

SD, standard deviation; COPD, chronic obstructive pulmonary disease; HPV, human papillomavirus.

### Incidence of pSS among patients with HPV infections

**Figure 2:**
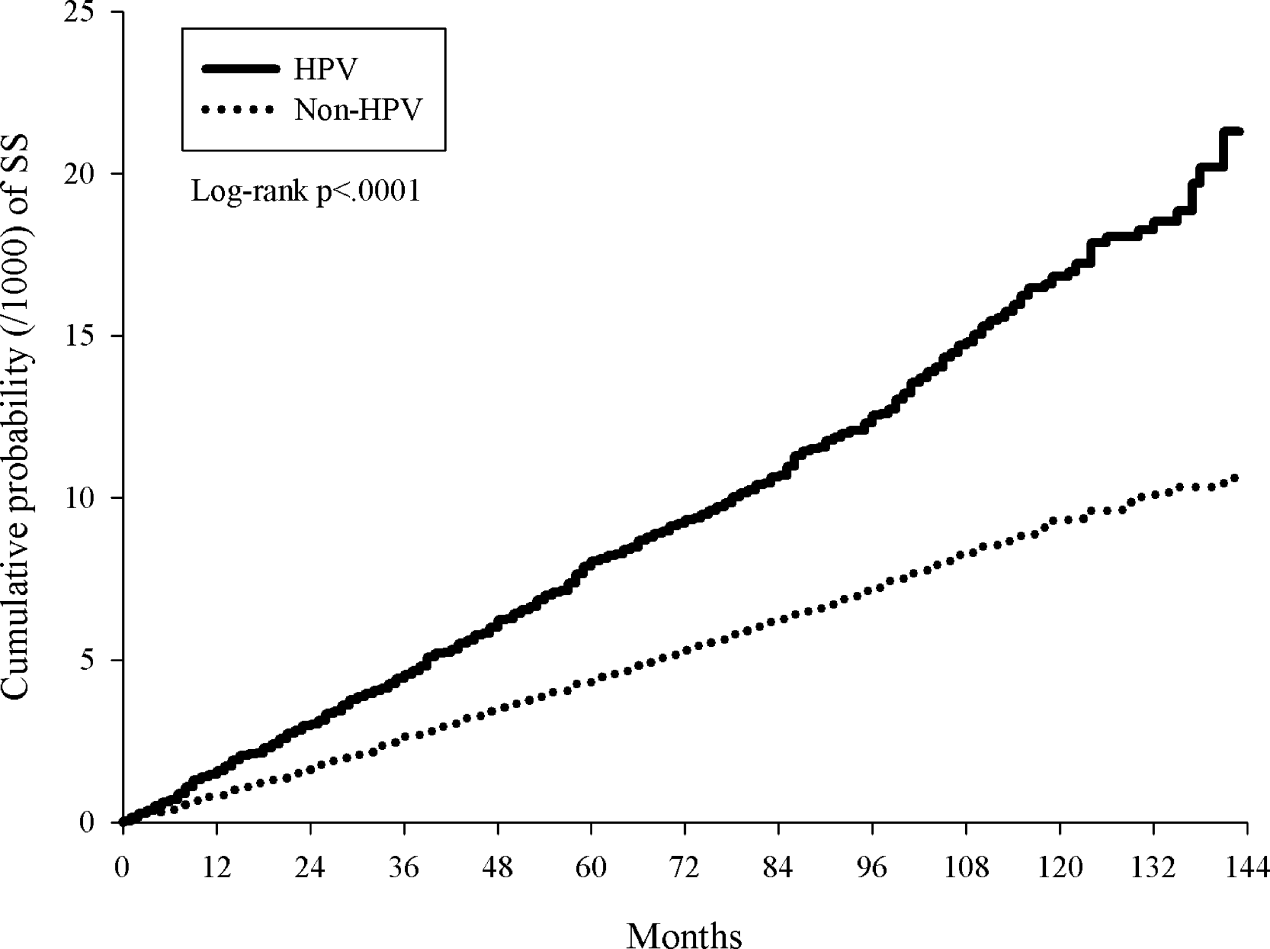
Kaplan-Meier curves of cumulative proportion of primary Sjogren’s syndrome in patients with HPV infections and non-HPV controls.

A total of 493 HPV patients and 1,081 controls were diagnosed with pSS, which corresponded to incidence rates of 13.61 and 7.53 per 100,000 person-months in the total follow-up period of 3,622,659 and 14,359,439 person-months for the HPV group and control group (**Table 2**). The pSS incidence rate in the HPV group was 1.81(95% CI=1.63-2.01) times higher than in the control group (**Table 2**). After almost 12 years of observation, the pSS cumulative incidence in the HPV group was significantly higher than in the control group (log-rank p<0.0001, **Figure 2**).

**Table 2:** Incidence of primary Sjogren’s syndrome.

	HPV infections	Non-HPV controls
	n = 47,300	n = 189,200
Follow up person-months	3,622,659	14,359,439
Event of Sjogren’s syndrome	493	1,081
Incidence rate* (95% C.I.)	13.61 (12.46-14.86)	7.53 (7.09-7.99)
IRR^†^	1.81 (1.63-2.01)	Reference

*per 10^5^ person-months.

^†^IRR, Incidence rate ratio.

### Risk for pSS in patients with HPV infections

Subsequent risk of pSS was estimated by using Cox proportional hazard regression (**Table 3**). Compared with the control group, patients with HPV exposure has a 1.64-fold aHR of pSS after adjusting for age, sex, and selected comorbidities (95% CI = 1.47–1.83) (**Table 3**). The risk of pSS increased with age by 2.64-fold (95% CI= 2.37-2.93) for over 45 years group (**Table 3**). Females has 2.78 times higher pSS risk than males (95% CI= 2.5-3.125) (**Table 3**). Patients with RA, COPD, pneumonia, dental caries, chronic liver disease, calculus of kidney, urinary tract infection, or arthralgia have increased the risk for pSS with aHRs of 3.67 (95% CI=2.86-4.70), 1.56 (95% CI=1.31-1.86), 1.29 (95% CI=1.00-1.67), 1.43 (95% CI=1.30-1.58), 1.32 (95% CI=1.14-1.53), 1.37 (95% CI=1.05-1.77), 1.29 (95%CI=1.14-1.46), and 1.31 (95% CI=1.14-1.49), respectively (**Table 3**).

**Table 3:** Risk factors for primary Sjogren’s syndrome in multiple Cox regression.

	aHR	95% C.I.	p value
HPV infections (reference: non - HPV controls)	1.64	1.47 - 1.83	<.0001
Age at baseline (reference: 16 - 45)			
≧45	2.64	2.37 - 2.93	<.0001
Sex (reference: females)			
Male	0.36	0.32 - 0.40	<.0001
Comorbidities (reference: no status of the comorbidities)			
Rheumatoid arthritis	3.67	2.86 - 4.70	<.0001
Pneumonia	1.29	1.00 - 1.67	0.0512
Bronchitis	0.91	0.70 - 1.19	0.4779
Dental caries	1.43	1.30 - 1.58	<.0001
Chronic liver disease	1.32	1.14 - 1.53	0.0002
Cholelithiasis	1.02	0.75 - 1.40	0.8814
Interstitial nephritis	1.07	0.79 - 1.45	0.6535
Calculus of kidney	1.37	1.05 - 1.77	0.0188
Urinary tract infection	1.29	1.14 - 1.46	<.0001
Arthralgia	1.31	1.14 - 1.49	0.0001
COPD	1.56	1.31 - 1.86	<.0001

aHR, adjusted hazard ratio; C.I., confidence interval; COPD, chronic obstructive pulmonary disease; HPV, human papillomavirus.

### Sensitivity analysis

After excluded individuals with at least 12 months of loss of follow-up, the aHR of pSS in HPV patients was 1.63 (95% CI=1.45-1.83) (**Table 4**). The subgroup analysis was conducted to determine which HPV subgroup was most susceptible to pSS. The risk of developing pSS in male HPV patients was higher than female patients (male HR=1.83, 95% CI=1.47-2.28 vs. female HR=1.58, 95% CI=1.40-1.79) (**Table 4**). The risks of having pSS were similar in all age groups. Patients between the age of 16 and 45 has HR of 1.60 (95% CI=1.34-1.91) and older than 45 years has HR of 1.67 (95% CI=1.46-1.97) (**Table 4**).

**Table 4:** Sensitivity analysis for the risk of primary Sjogren’s syndrome following HPV infections.

	HR	95% C.I.	p value
Restriction			
Excluded the individuals cannot be tracked for at least 12 months (239 patients with HPV infections and 1,609 non - HPV controls were excluded)	1.63	1.45 - 1.83	<.0001
Subgroup analysis stratified by sex			
Female	1.58	1.40 - 1.79	<.0001
Male	1.83	1.47 - 2.28	<.0001
p for interaction			0.1499
Subgroup analysis stratified by age at baseline			
Aged16 - 45 years old	1.60	1.34 - 1.91	<.0001
Aged ≧45 years old	1.67	1.46 - 1.91	<.0001
p for interaction			0.8251

HR, hazard ratio; C.I., confidence interval; HPV, human papillomavirus.

## Discussion

The etiology of pSS is still inconclusive. Currently, the genetic background, the environmental stimulation on epigenetics, and the use of immunotherapeutic agents (36–40) are proposed to trigger pSS. However, the evidence for the underlying mechanism is still insufficient. Out of all the external factors, viral infection is suspected to be the main risk. Several studies have suggested the possible role infections play in the development of pSS. To date, no studies have clarified the association between HPV infections and pSS. This paper demonstrates epidemiological evidence in favor of a correlation by using nationwide, population-based data in Taiwan. In this study, patients with HPV exposure were associated with increased pSS incidence. RA, COPD, pneumonia, dental caries, calculus of kidney, urinary tract infection, and chronic liver disease also increased pSS incidence.

During the infected stage, the HPV deoxyribonucleic acid (DNA) in the infected cells may activate T cells or B cells through MHC pathways (41, 42). Majewski and Jablonska reported HPV could serve as super antigens to activate polyclonal T cells, which could trigger autoimmune phenomenon of psoriasis (13). The specific sequence of a certain type of HPV gene that codes for viral protein, which acts as autoantigens, need further research to identify. A previous epidemiological study in Taiwan found the association between HPV infection and psoriasis onset. In this study, pSS patients were prominent for the risk of psoriasis through activation of T cells, as similar to the reported associations between bacterial (43–47) or viral (48) infections in other autoimmune diseases.

In Taiwan, pSS is one of the catastrophic illness specified by the National Health Insurance program. Attending physicians must submit related clinical information including pSS patient histories, laboratory, and pathological data to the National Health Insurance Administration (NHIA) to apply for a catastrophic illness certificate (CIC). A committee under the NHIA would review all applications in according to the criteria of the American-European Consensus Group (AECG) for pSS (49). Patients must meet either 4 out of 6 AECG criteria including No.4 (Histopathology) or No.6 (Autoantibodies), or 3 out of 4 objective criteria including No.3, No.4, No.5, No.6. These features recognized by AECG could reflect the immune-pathogenesis of patients’ adaptive immune systems.

There are limitations in this study. Firstly, the diagnoses of HPV infections may not be fully accurate. About 75% of females with history of sexual intercourse were susceptible to HPV infections, but the infection symptoms were not always clinically identifiable in patients with HPV infections, which could cause an underestimated rate of HPV-infected patients in this survey. Secondly, the diagnosis of HPV infection in Taiwan mainly depended on pap smear methods and viral DNA types analysis. Smear tests were convenient, but their like-hood ratios (LRs) of HPV infections were as low as 50%. Southern blot analysis, PCR, and other molecular level laboratory analysis were usually performed to increase the true positive rates and to identify specific type of HPV DNA (41). Furthermore, most physicians would not detect HPV antigens in pSS patients’ lesions through biopsy. Therefore, the imperfect decision-making process could result in information bias in random. Thirdly, data on specific clinical subsets of pSS triggered by HPV infections that included data on Schirmer’s test, history of HPV vaccinations and medications including steroids or other drugs for pSS, and subsequent mortality of participants, were not included in this study, which may be investigated in future studies to support findings of the present study.

Our study employed a large, nationwide sample with high external validity to neutralize deviation from selection bias. In the future, more clinical data are required to reflect a more accurate disease course of HPV infections. Genetic or cytological analyses of lesions that specify HPV genotypes and phenotypes of the infections and biomarkers with high LR values are key prognosis factors of HPV infections, which may solidify our knowledge on the relationship between HPV infections and the risk of pSS. On the other hand, this would help with future disease screenings, patient educations (50), and early treatments to be implemented.

## Conclusion

In this population-based cohort study, patients with HPV infections presented with significantly higher risk of new-onset pSS. RA, COPD, pneumonia, dental caries, chronic liver disease, calculus of kidney, urinary tract infection, and arthralgia were also independent risk factors for pSS.

## Data availability statement

The original contributions presented in the study are included in the article/supplementary material. Further inquiries can be directed to the corresponding authors.

## Author contributions

H-HC and KM contribute to conception and design of the study. KM and W-JC organized the database and performed the statistical analysis. H-HC wrote the first draft of the manuscript. KM and CD wrote sections of the manuscript. All authors contributed to manuscript revision, read and approved the submitted version.

## Conflict of interest

The authors declare that the research was conducted in the absence of any commercial or financial relationships that could be construed as a potential conflict of interest.

## Publisher’s note

All claims expressed in this article are solely those of the authors and do not necessarily represent those of their affiliated organizations, or those of the publisher, the editors and the reviewers. Any product that may be evaluated in this article, or claim that may be made by its manufacturer, is not guaranteed or endorsed by the publisher.
