# Corrigendum: Risk of primary Sjogren’s Syndrome following human papillomavirus infections: a nationwide population-based cohort study

**DOI:** 10.3389/fimmu.2024.1470841

**Published:** 2024-08-08

**Authors:** Huang-Hsi Chen, Kevin Sheng-Kai Ma, Chen Dong, Wen-Jung Chang, Kuan-Rong Gao, Wuu-Tsun Perng, Jing-Yang Huang, James Cheng-Chung Wei

**Affiliations:** ^1^ Division of Allergy, Immunology, and Rheumatology, Chung Shan Medical University Hospital, Taichung, Taiwan; ^2^ Institute of Medicine, Chung Shan Medical University, Taichung, Taiwan; ^3^ Center for Global Health, Perelman School of Medicine, University of Pennsylvania, Philadelphia, PA, United States; ^4^ Department of Epidemiology, Harvard T.H. Chan School of Public Health, Boston, MA, United States; ^5^ Graduate Institute of Biomedical Electronics and Bioinformatics, National Taiwan University, Taipei, Taiwan; ^6^ Department of Obstetrics and Gynecology, Dajia Lees General Hospital, Taichung, Taiwan; ^7^ Department of Obstetrics and Gynecology, Yuanli Lees General Hospital, Miaoli, Taiwan; ^8^ National Pingtung University of Science and Technology, Department of Recreational Sport & Health Promotion, Pingtung, Taiwan; ^9^ Institute of Medicine, College of Medicine, Chung Shan Medical University, Taichung, Taiwan; ^10^ Graduate Institute of Integrated Medicine, China Medical University, Taichung, Taiwan

**Keywords:** Human papillomavirus, infection, autoimmunity, primary Sjogren’s Syndrome, cohort study

In the published article, there was an error regarding the affiliations for authors Jing-Yang Huang and James Cheng-Chung Wei. Affiliation 8 has been removed from their affiliations.

In the published article, there was an error in affiliation 8. Instead of “Center for Health Data Science, Chung Shan Medical University Hospital, Taichung, Taiwan”, it should be “National Pingtung University of Science and Technology, Department of Recreational Sport & Health Promotion, Pingtung, Taiwan”.

The authors apologize for these errors and state that they do not change the scientific conclusions of the article in any way. The original article has been updated.

